# Recent Advances and Challenges in Anion Exchange Membranes Development/Application for Water Electrolysis: A Review

**DOI:** 10.3390/membranes14040085

**Published:** 2024-04-05

**Authors:** Lu Liu, Hongyang Ma, Madani Khan, Benjamin S. Hsiao

**Affiliations:** 1 State Key Laboratory of Organic-Inorganic Composites, Beijing University of Chemical Technology, Beijing 100029, China; 2 Department of Chemistry, Stony Brook University, Stony Brook, NY 11794-3400, USA

**Keywords:** water electrolysis, anion exchange membrane, ion conductivity, alkaline stability, patents

## Abstract

In recent years, anion exchange membranes (AEMs) have aroused widespread interest in hydrogen production via water electrolysis using renewable energy sources. The two current commercial low-temperature water electrolysis technologies used are alkaline water electrolysis (AWE) and proton exchange membrane (PEM) water electrolysis. The AWE technology exhibited the advantages of high stability and increased cost-effectiveness with low hydrogen production efficiency. In contrast, PEM water electrolysis exhibited high hydrogen efficiency with low stability and cost-effectiveness, respectively. Unfortunately, the major challenges that AEMs, as well as the corresponding ion transportation membranes, including alkaline hydrogen separator and proton exchange membranes, still face are hydrogen production efficiency, long-term stability, and cost-effectiveness under working conditions, which exhibited critical issues that need to be addressed as a top priority. This review comprehensively presented research progress on AEMs in recent years, providing a thorough understanding of academic studies and industrial applications. It focused on analyzing the chemical structure of polymers and the performance of AEMs and established the relationship between the structure and efficiency of the membranes. This review aimed to identify approaches for improving AEM ion conductivity and alkaline stability. Additionally, future research directions for the commercialization of anion exchange membranes were discussed based on the analysis and assessment of the current applications of AEMs in patents.

## 1. Introduction

The globally escalating energy demand, exponential growth in greenhouse gas emissions, and the gradual depletion of fossil fuels constitute one of humanity’s most formidable challenges [1]. Hydrogen, a “zero-carbon emission” renewable resource, achieves efficient conversion between electrical and hydrogen energy via water electrolysis technology. Simultaneously, it overcomes the instability and intermittency of solar and wind power generation, enabling large-scale energy conversion and storage. Therefore, the electrolysis of water for hydrogen production using renewable resources is attracting widespread interest [2,3,4]. Hydrogen plays a crucial role in human industrial life, continuously increasing global demand for hydrogen gas, with a current production that exceeds 70 million tons per year. However, over 95% of hydrogen is produced via steam methane reforming (SMR), resulting in substantial carbon dioxide emissions and severe environmental pollution [5,6].

Alkaline water electrolysis (AWE) was first commercially available in the 1970s and was regarded as the only established technology for hydrogen production. Nevertheless, PEM water electrolysis was developed rapidly and recently moved into a primary commercial stage. The conventional separator used for AWE (i.e., the diaphragm) was woven porous polyphenylene sulfide (PPS) fabrics, governed by Toray in the market, and was outstanding with high resistance against highly concentrated alkaline aqueous solutions (e.g., 6 M KOH) at a temperature of above 110 °C for about eight years. The advantages of AWE water electrolysis are long-term stability and low cost for the chosen separator and catalyst. However, the high hydrogen production efficiency (>70%, achieving 75% with a 25 μm thick Nafion212 membrane [7]) demands high air resistance and ionic conductivity, promoting the development of PEM membranes in water electrolysis.

Proton exchange membrane (PEM) water electrolysis offers high hydrogen production efficiency by replacing the porous separator with a nonporous PEM membrane where H^+^ is transported through the membrane via an ion exchange mechanism. The air resistance of the membrane was enhanced by its nonporous structure, which served as an electrolyte instead of 6 M KOH in AWE and was swollen in an aqueous environment. However, the disadvantage of the PEM is mainly due to the membrane materials where only perfluorosulfonic acid (PFSA) could be used to fabricate the membrane, which is not economically sustainable.

Therefore, green energy conversion devices with increased cost-effectiveness (the cost of nickel-based catalysts being one-thousandth and eight-thousandth of platinum and iridium catalyst costs, respectively [8]) and high hydrogen efficiency (>65%) [9] such as anion exchange membrane water electrolysis in addition to AWE and PEM devices, driven by renewable energy sources, have received significant attention and booming drastically which were reviewed comprehensively [10].

### 1.1. Water Electrolysis Technology

Water electrolysis technology is a technique that converts water into hydrogen and oxygen gases using electricity at a relatively low temperature. It is an electrochemical water-splitting technology that enables zero-emission hydrogen production [11]. The basic reaction of water electrolysis is represented by Equation (1).

(1) $$H_{2}O+Electricity\;\left(237.2\;KJ{mol}^{-1}\right)+Heat\;\left(48.6\;KJ{mol}^{-1}\right)\to H_{2}+\frac{1}{2}O_{2}$$

Depending on the method of electrolyte used, electrolysis cells can be classified into three common types: alkaline water electrolysis, PEM water electrolysis, and anion exchange membrane (AEM) water electrolysis [12].

### 1.2. Alkaline Water Electrolysis

**Figure 1:**
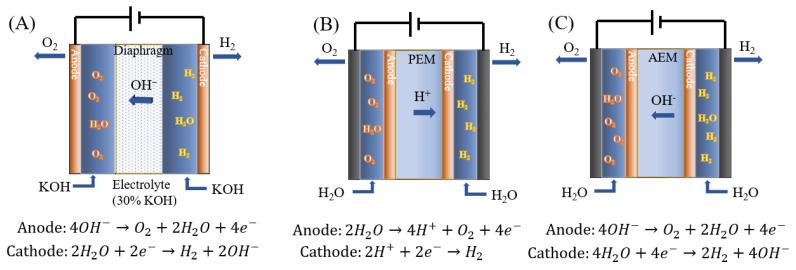
Schematic diagram of electrolysis cells: (**A**) AWE, (**B**) AEM water electrolysis, and (**C**) PEM water electrolysis, respectively [13,14,15].

The principal layout of an AWE is shown in Figure 1A.

Two metal-based electrodes (Ni and Fe) are immersed in the electrolyte (20–30% KOH solution) separated by a membrane [16]. The circulating KOH electrolyte provides the necessary alkaline environment. The porous membrane serves as a separator to isolate the cathode and anode while facilitating the conduction of OH^-^ and preventing the gas crossover.

AWE is a well-known commercialized low-temperature electrolysis technology that does not require platinum-based metal catalysts. In these systems, the membranes are typically made out of porous materials, including ceramic oxides such as asbestos and potassium titanate or polymers like polypropylene and polysulfide [17]. These materials are cost-effective, and their hydrogen can achieve up to 99% purity. However, membranes suffer from drawbacks such as poor gas tightness, high surface resistance, low hydrogen production efficiency (59–70%), and poor alkaline stability [12,18,19]. These deficiencies significantly reduce the performance of the electrolysis cell. Therefore, the preparation of AEM meets production standards, but like many other systems, it still has room for improvement.

### 1.3. PEM Water Electrolysis

In contrast to AWE, PEM water electrolysis has advantages such as high ion conductivity (0.1 S/cm), low ohmic losses (maximum achievable current density approximately 2 mA/cm^2^), and minimal gas crossover. This technology is relatively mature and widely applied, but it is still on the way to being commercialized [18]. The basic setup of PEMWE is illustrated in Figure 1B, where a PEM (i.e., Nafion membrane) separates the two half-cells, and the electrodes are typically directly mounted on the membrane.

However, the corrosive acidic environment provided by the proton exchange membranes increases the cost of PEMWE due to the demand for precious metal catalysts made of iridium and platinum [14]. Additionally, the reliance of proton exchange membranes on fluorinated polymers leads to the emission of fluorocarbon gases during their production, causing severe environmental impact [20].

### 1.4. AEM Water Electrolysis

AEM water electrolysis has been extensively researched as an alternative approach to tackle the challenges with AWE and PEM mentioned above. They allow the use of non-precious metal catalysts, and AEMs are cost-effective, significantly reducing production costs. Additionally, AEMs exhibit good gas tightness and eliminate gas crossover, resulting in hydrogen purity as high as 99.99%, making them a key component determining the performance of the electrolyzer [21].

Therefore, AEMWE represents a low-temperature water electrolysis technology that combines the advantages of both AWE and PEMWE [14,22]. The basic layout is depicted in Figure 1C.

The main components of an AEMWE cell include the anion exchange membrane (AEM), gas diffusion layer, electrocatalysts, and current collector [23].

The electrolysis of water consists of two separate half-cell reactions, including the hydrogen evolution reaction (HER) at the cathode and the oxygen evolution reaction (OER) at the anode. In the HER process, the sluggish kinetics of OER is the primary factor limiting the overall water electrolysis performance. To reduce overpotential, besides developing highly active catalysts for both HER and OER, AEMs need to exhibit excellent ion conductivity [24].

In contrast, PEM possesses advantages such as high hydrogen production efficiency, high ion conductivity, excellent gas tightness, and stability. However, PEM based on perfluorosulfonic acid resin is costly. Therefore, PEMWE cannot be widely used for large-scale, global hydrogen production [14]. Most importantly, PEMWE relies on fluorinated polymers and emits fluorocarbon gases during production, causing significant environmental impact [20]. As a low-temperature water electrolysis technology that combines the advantages of AWE and PEMWE, AEMWE has proven to be more efficient and can operate without noble metal catalysts in a less alkaline electrolyte environment, providing critical advantages. However, AEMWE is still an evolving technology, and efforts are needed to narrow the efficiency and lifespan gap compared to PEMWE before commercialization [24,25].

Due to the higher stability of PEM, PEMWE exhibits a longer lifespan (up to 5000 h), a hydrogen production efficiency of 57%, a high current density of 1–3 A/cm^2^, and a lower area-specific resistance (approximately 68 mΩ∙cm^2^). The primary challenge for the current PEMWE is reducing production costs. Although AWE is a well-known technology with a hydrogen production efficiency close to 60%, it still faces challenges that cannot be overcome at present, such as a low maximum current density (0.2–0.4 A/cm^2^), difficulty operating under high-pressure differentials (1–30 bar, whereas PEMWE operates at 30–76 bar), and slow response times [18,26,27,28].

AEMWE technology is relatively new, and to achieve widespread application, several key issues need to be addressed, such as long-term stability and high current density (currently 0.2–1 A/cm^2^) [29]. Although many AEMs have achieved an ion conductivity of 0.1 S/cm at 60–80 °C, the durability of AEMs at this temperature (less than 1000 h) remains a significant challenge [5]. Until recently, single-cell AEMWEs could operate for thousands of h at 60 °C and a current density of 1 A/cm^2^. Currently, AEMs must meet a minimum ion conductivity target of 0.1 S/cm and an area-specific resistance target of less than 70 mΩ∙cm^2^ [26].

Some challenges remain for AEMs: (1) overcoming the alkaline stability limitations of most AEMs; (2) reducing the area-specific resistance of AEMs; and (3) further increasing ion conductivity.

**Figure 2:**
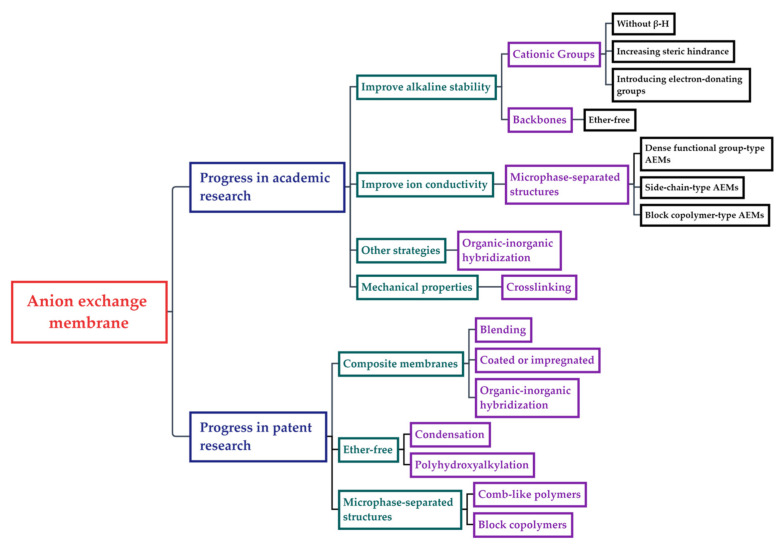
Mind mapping of anion exchange membranes.

Therefore, a comprehensive analysis of AEM will be conducted in this review, ranging from molecular engineering to in situ performance evaluation, mainly based on the research directions revealed by the anatomy of currently available patents, to outline the future development of AEM considering the existing obstacles (Figure 2).

## 2. Progress in Academic Research of Anion Exchange Membrane

**Figure 3:**
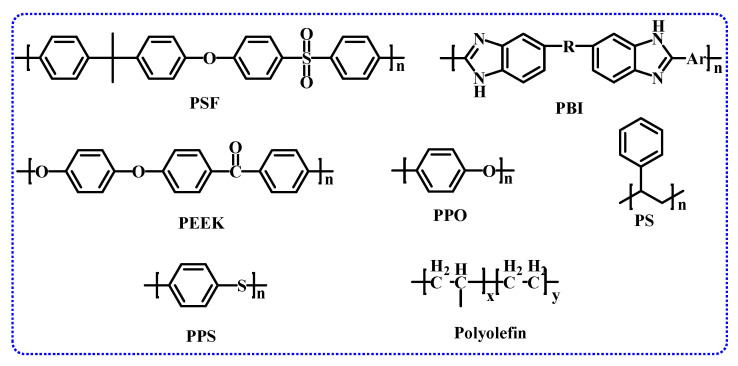
Main polymer chains commonly used in AEMs (Ar = phenyl).

AEM comprises a polymer backbone and cationic functional groups, serving as the core of AEMWE systems and a crucial component determining the performance and lifespan of AEMWE [30]. The polymer backbone’s performance dictates AEMs’ mechanical and thermal stability, requiring good membrane-forming ability, excellent mechanical and chemical properties, and high alkaline resistance to meet preparation and usage demands. Typical polymer backbones include polybenzimidazole (PBI) [31,32], polyether ether ketone (PEEK) [33], polysulfone (PSF) [34,35,36], polystyrene (PS) [37,38,39], polyphenylene ether (PPO) [32,40,41,42], and polyolefins [43], as shown in Figure 3.

**Figure 4:**
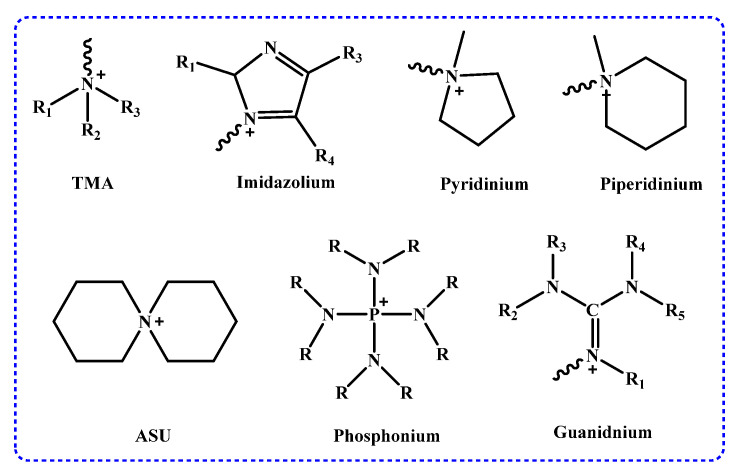
Functional cationic groups commonly used in AEMs (R = H, alkyl or phenyl).

Common cationic functional groups include trimethylamine (TMA) [31,38], imidazolium (TMI) [32,44], pyridinium (PYR), piperidinium (PIP) [45,46], quaternary ammonium groups [47], 6-Azonia-spiro[5.5]undecane (ASU) [48,49,50,51], and quaternary phosphonium [52], which offer the function of anion exchange to the AEM via electrostatic interaction in water electrolysis, as shown in Figure 4.

In a water electrolysis cell, AEM plays a role in ion conduction while preventing the crossover of hydrogen and oxygen. The ideal characteristics for AEM required in water electrolysis cells include high OH^-^ conductivity, long-term alkaline stability, low dimensional expansion, and the ability to prevent gas crossover [21].

Over the past decade, research has predominantly focused on other traditional water electrolysis technologies, such as AWE, with relatively less emphasis on AEMWE due to the major concern regarding the stability of the AEMs. Additionally, due to the lower OH^-^ migration rate, the ion conductivity of AEM is much lower than that of PEM. Most AEMWE exhibits a sharp decline in performance after extended operation periods [49,53] because of the degradation of AEM under alkaline conditions; even commercial AEMs fail to achieve long-term durability exceeding 3000 h at 1 A/cm^2^ [23].

The historically lower ion conductivity and alkaline stability of AEM have been significant barriers to the commercialization of AEMWE. Hence, there is an urgent need to develop AEMs with high alkaline stability and ion conductivity for water electrolysis. Researchers are exploring methods to enhance alkali stability by delving into degradation mechanisms.

### 2.1. Degradation Mechanisms

**Figure 5:**
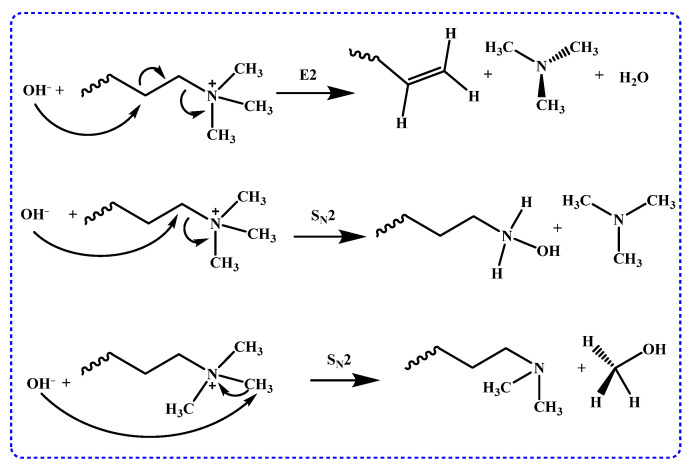
Mechanism of Hofmann elimination and nucleophilic substitution degradation of quaternary ammonium groups.

Cationic functional groups in AEMs have been extensively studied, with current research primarily focusing on quaternary ammonium (QA) groups. QA salts, like trimethylalkylammonium, exhibit good ion conductivity and are easy to synthesize in AEMs. The degradation of QA groups in alkaline environments is mainly attributed to Hofmann elimination and OH^-^ attacking N-alkyl via nucleophilic attack (S_N_2 substitution reaction), as illustrated in Figure 5 [15,54,55].

It was systematically studied on the alkaline stability of many different QA cations under the same conditions (e.g., 10 M NaOH solution, 160 °C). The alkaline stability order obtained was ASU > N-methylpiperidinium (MPIP) ≈ N-methylpyrrolidinium (MPY) > TMA > TMI. Except for a few cations, most QAs exhibited good alkaline stability. Among them, piperidine-based ASU had the highest half-life. At the same time, imidazolium TMI showed the poorest alkaline stability [56], probably due to the inherent ring tension of pyridinium cations, which makes them exhibit high resistance under high-temperature and alkaline conditions. The stability is further reduced when there are heteroatoms or other electron-withdrawing groups. Due to the almost complete lack of spatial shielding or substituents with an electron-inductive effect, aromatic-based QA has the fastest decomposition rate. This result has also been confirmed in other studies [57].

In addition to cationic functional groups, the structure of the polymer backbone is another crucial factor in assessing the alkaline stability of AEM. Among various reported polymer backbones, those containing aromatic ether groups (such as PPO, PSF, PEEK, etc.) have garnered significant attention due to their excellent overall performance, ease of preparation, and good mechanical properties [58,59,60,61].

**Figure 6:**
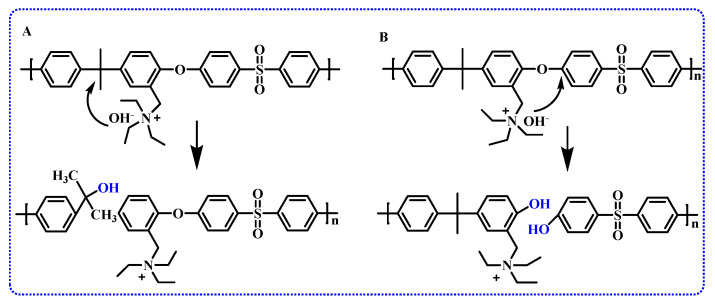
The hydrolysis of (**A**) quaternary carbon and (**B**) ether bonds in PSF AEMs containing relatively stable fixed cations [62].

In another study, the degradation mechanisms of anion exchange membranes based on PSF backbones have been investigated. It was proposed that functionalization at the benzyl position of polysulfone leads to the exposure of the polymer backbone to alkaline solutions, resulting in the hydrolysis of quaternary carbon and ether bonds. Figure 6 illustrates these two degradation mechanisms. Therefore, it is speculated that connecting the cationic groups to the PSF backbone using alkyl chains can enhance stability [62].

Polymer backbones with aromatic ether groups, such as polysulfone and polyarylether, accelerate the degradation of the main chain due to the introduction of C-O bonds. However, C-O bonds allow the polymer backbone to rotate freely, ensuring excellent mechanical properties and solubility. Therefore, polymers containing C-O bonds remain the most used polymer backbones even though there is a problem of alkaline instability.

Due to the awareness of the susceptibility of polymer backbones containing aromatic ether groups to degradation in alkaline environments, many researchers shifted their focus on polymer backbones without aromatic ether, such as polyolefins, PS, or the synthesis of non-aromatic ether polymer backbones via coupling [63], addition [64], acid-catalyzed Friedel–Crafts hydroalkylation [65], and other reactions.

### 2.2. Strategies to Improve AEM Alkaline Stability

Currently, various types of AEMs have been developed, and progress in enhancing alkaline stability has driven the advancement of AEMWE [66]. The attempts were focused on both the cationic functional groups and polymer backbones, considering the degradation mechanism, i.e., β-H elimination and S_N_2 substitution.

#### 2.2.1. Cationic Functional Groups

Methods to improve the alkaline stability of cationic functional groups include (1) designing structures without β-H to inhibit Hofmann elimination [67,68,69]; (2) increasing steric hindrance around the cationic functional groups to protect AEM from attack by OH^-^ [70,71]; and (3) introducing electron-donating groups near the cationic functional groups to prevent their vulnerability to OH^-^ attack due to electron deficiency [72,73,74].

**Figure 7:**
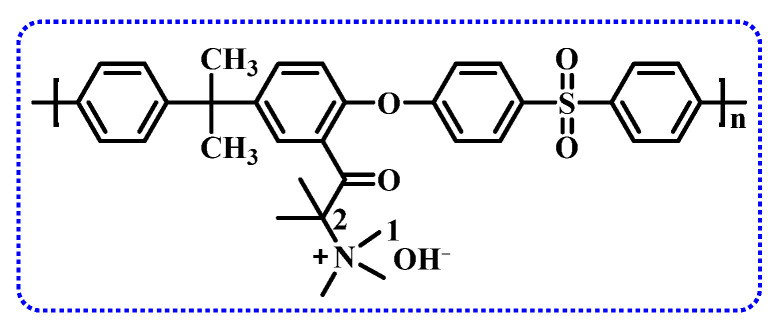
Structure of β-H-free polysulfone AEM.

The Hofmann elimination is the most likely the major degradation pathway for cationic functional groups [75]. Therefore, one promising method to enhance alkaline stability is to design structures without or with the least amount of β-H to inhibit Hofmann elimination, thereby improving the stability of cationic functional groups. A good example is shown in the structure in Figure 7. Due to the elimination of β-H, the polysulfone AEM with this structure maintains unchanged ion conductivity after soaking in a 1 M NaOH solution at 80 °C for 5 days, and after 10 days, the conductivity remains at 95% of the original level [76].

While enhancing the alkaline stability of AEM by substituting β-H can offer some improvement, degradation can still occur due to other mechanisms, such as the previously mentioned S_N_2 substitution. This involves a direct nucleophilic attack by OH^-^ on the nitrogen atom of the quaternary ammonium group and the degradation of the polymer backbone [5]. It is indicated that improving the alkaline stability of cationic functional groups is more beneficial for enhancing AEM alkaline stability compared to the stability at the C2 position [77]. Therefore, increasing the steric hindrance around cationic functional groups has also become an effective approach to enhance the stability of QA.

**Figure 8:**
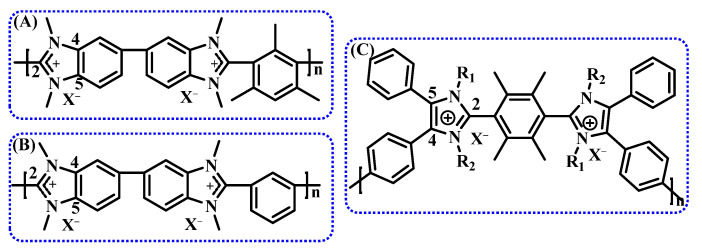
Polybenzimidazole with spatial protection (**A**,**B**), novel polyaromatic imidazolium compound (**C**).

Taking imidazolium as an example, benzyl-protected benzimidazolium cations at the C2 position (structures A and B in Figure 8) exhibit excellent alkaline stability (i.e., half-life > 5000 h). The enhanced steric hindrance at the C2 position of the benzimidazolium group hinders nucleophilic attack by OH^-^, effectively inhibiting ring-opening degradation [78]. The benzimidazolium protected by structure B remains stable for an extended period in a 1 M hydroxide solution at 80 °C but degrades more rapidly under high-temperature and highly corrosive conditions. After soaking in a 5 M NaOH solution at 80 °C for one week, a 60% degradation is observed [79]. To explore this phenomenon, the impact of substituent characteristics and positions on the chemical stability of imidazolium cations was systematically evaluated. It is found that substituent characteristics and positions significantly influence the overall stability of cations. Specifically, imidazolium cations with substitutions at the C2 position can effectively inhibit ring-opening degradation, with the most effective being the 2,6-dimethylphenyl substitution. Methyl or phenyl substitutions at the C4 and C5 positions further enhance the stability of cationic groups [75,80].

A novel polyaromatic imidazolium compound with spatial protection (structure C in Figure 8) was reported recently. After soaking in 10 M KOH at 80 °C for 240 h, the imidazolium group maintained 97.7%, with a half-life of 8000 h, showcasing outstanding stability. The stability increases with the length of the N-alkyl chain in the molecular structure. However, due to the reduced water content, the energy barrier for ion transport increases, resulting in an ion conductivity of only 12 mS/cm at 80 °C [69].

In summary, increasing steric hindrance can effectively enhance the alkaline stability of AEMs, where multiple substituents prevent nucleophilic substitution and ring-opening degradation. However, this concurrently reduces the ion conductivity of OH^-^. Since most studies on imidazolium substitution patterns are typically limited to commercially available imidazolium, it is necessary to develop new synthetic routes to attach alkali-stable novel imidazolium cations to polymers.

Introducing electron-donating spacer groups in the side chain can increase the electron cloud density around the benzyl carbon, exhibiting a higher energy barrier for the degradation of the QA group and thus enhancing the alkaline stability of the cationic functional group [79]. N-spirocyclic quaternary ammonium-functionalized side chains with flexible ether spacers via the Williamson reaction were synthesized. The constrained-ring conformation of the N-spirocyclic cation and the electron-donating effect of the ether spacer endowed the polyaromatic imide AEM with excellent alkaline stability. After immersing in 1 M KOH solution at 80 °C for 720 h, the conductivity remained at 95.6% of the original value, significantly improving the alkaline stability of the AEM [81]. Additionally, introducing flexible ether bonds in the side chain promoted the aggregation of N-spirocyclic cations, resulting in a high OH^-^ ion conductivity (85.7 mS/cm at 80 °C). Furthermore, the alkaline stability of polyphenyl ether AEM with flexible alkyl side chains constructed via the Suzuki reaction was confirmed. After immersing the AEM in 1 M NaOH solution at 60 °C for 168 h, the OH^-^ conductivity remained around 90% of the original conductivity [82].

It is a new approach to enhancing the alkaline stability of cationic functional groups that involves using cyclic cations as quaternary ammonium groups. These cyclic ammonium cations still primarily degrade via nucleophilic substitution via an opening mechanism under alkaline conditions. However, due to their ring tension, five-membered rings exhibit higher alkaline stability than six-membered and seven-membered rings. This is because, in general, larger rings tend to degrade via Hofmann elimination reactions, while smaller rings degrade only via opening substitution reactions [83].

It is foreseeable that the integrated application of these strategies is expected to improve the stability of anion exchange membranes under alkaline conditions, thereby promoting the development of electrolysis technologies.

#### 2.2.2. Polymer Backbones

As discussed before, anion exchange membrane (AEM) alkaline stability is primarily determined by the cationic functional groups and the polymer main chain. In addition to the type and structure of cations, the composition of the polymer backbone is crucial, greatly affecting AEMs’ mechanical and chemical stability [84]. It is found via density functional theory (DFT) calculations and in situ degradation experiments that AEMs based on polyarylether exhibit lower stability compared to those without ether in the polyarylene backbone [85]. Therefore, for polymer main chains, ether-free aromatic backbones are the preferred choice in structural design. Examples include polyarylene [63,86], polyfluorene [86,87], and polyolefin-type polymers [36,88], which have been extensively studied and demonstrate excellent chemical and thermal stability.

**Figure 9:**
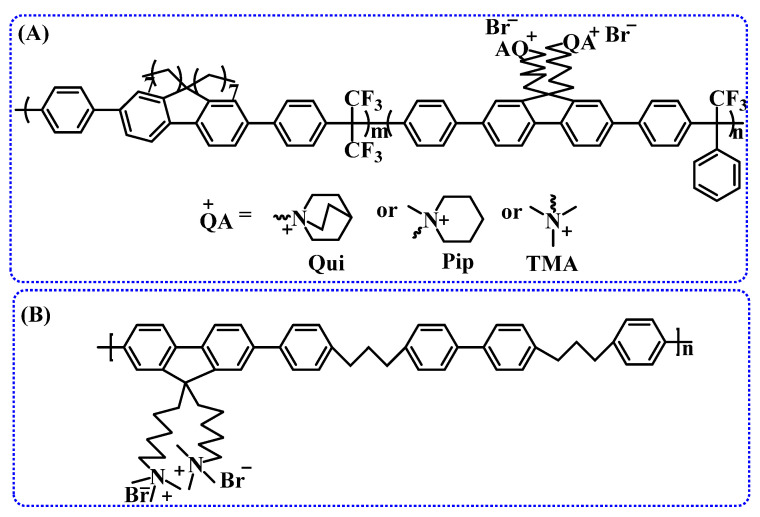
Structures of poly (arylene ether) main chains prepared by polycondensation based on Friedel–Crafts (**A**) and Suzuki cross-coupling (**B**) reactions, respectively.

Therefore, in designing durable and high-performance polymers, it is essential to consider the physical and chemical properties of the polymer’s main chain [89]. Friedel–Crafts condensation is a common synthetic method used to prepare ether-free main chains. A polyfluorene main chain (Structure A in Figure 9) via Friedel–Crafts condensation and connected to quinuclidine ring cations using a hexyl spacer was synthesized and explored the effects of polymer main chain on the alkaline stability of the AEM. This AEM exhibits excellent alkaline stability, showing no evidence of any ring-opening degradation after 672 h in a 2 M NaOH solution at 80 °C, with less than 2% Hofmann elimination [90].

Moreover, a polyfluorene-based polymer without aromatic ethers using the Suzuki cross-coupling reaction was fabricated, which has pendant ammonium groups on the side chain and propyl spacers on the main chain, as shown in Structure B in Figure 9. Introducing propyl spacers into the main chain enhances the flexibility of the polymer and allows it to form ion clusters effectively. This AEM exhibits not only good alkaline stability (80 °C, 1 M KOH, 720 h) but also excellent ion conductivity (122 mS/cm, 80 °C) [91].

Additionally, ion-solvating membranes are gaining increasing attention as another effective method to enhance alkaline stability in the field of water electrolysis [92,93,94]. An article published in 2023 demonstrated that ion-solvating membranes, prepared from alkali-stable ammonium network precursors and ion-solvating polymer matrices, exhibit excellent alkaline stability. Under conditions of 70 °C and 1 M KOH, the alkaline stability exceeds 300 h, as shown in the article. Short-term durability tests also indicate superior durability compared to commercial AEMs [95]. Excellent alkaline stability and ion conductivity of ion-solvating membranes based on PBI polymers were also confirmed in another study. After immersion in an 80 °C, 8 M KOH solution for 1000 h, the conductivity of the PBI anion exchange membrane remained at 89% of the initial conductivity [96]. Therefore, ion-solvating membranes represent one of the effective methods for preparing long-lasting AEMs.

Therefore, various methods have been employed to synthesize aryl-based main chains without ethers to overcome the instability issue of main chains containing electron-withdrawing groups under alkaline conditions. These methods include acid-catalyzed Friedel–Crafts condensation [94], Diels–Alder reaction [97], and metal-catalyzed coupling reactions [63], contributing significantly to the improvement in alkaline stability in AEMs.

### 2.3. Ion Conductivity of AEM

Various anion exchange membranes for water electrolysis were designed and fabricated to improve the ion conductivity of the membranes. Most of these membranes are main-chain-type AEMs, where cationic functional groups are directly and randomly distributed along the polymer backbone. However, due to the constraints imposed by the polymer main chain, the movement space of ion exchange groups is limited, preventing the formation of an effective aggregated structure and resulting in lower ion conductivity [98,99].

AEMs’ high ion conductivity can be achieved by increasing the ion exchange capacity (IEC); however, a high IEC leads to elevated water uptake and swelling, consequently reducing the mechanical strength of the membrane. Research indicates that microphase-separated AEMs offer a promising approach to developing AEMs with high conductivity and low swelling [100]. The hydrophilic regions form continuous ion transport channels, facilitating OH^-^ transport, while the hydrophobic regions effectively suppress excessive membrane swelling [101].

Currently, there are three main types of AEM structures that can self-assemble into well-defined microphase-separated structures: dense functional group-type AEMs, side-chain-type AEMs, and block copolymer-type AEMs.

For dense functional group-type AEMs, the cationic functional groups are typically concentrated in a specific polymer segment or structural unit, forming hydrophilic ionic clusters that induce the formation of hydrophilic/hydrophobic microphase separation. This establishes connected ion transport channels, enhancing ion conductivity [98]. To achieve a dense functional group arrangement, monomers containing functionalized groups are often polymerized and then modified to create segments or structural units with a high density of cationic functional groups. A monomer with six methyl groups was introduced into a polysulfone backbone via condensation reactions, creating an anion exchange membrane with pendant imidazolium groups. The locally high functional group density of such membranes facilitates the aggregation of groups, promoting the formation of microphase-separated structures. Results showed that a membrane with an ion exchange capacity (IEC) of 2.2 meq/g exhibited an ion conductivity of 29 mS/cm at 60 °C. While this represents an improvement over non-dense AEMs, the ion conductivity is still relatively low, and the alkaline resistance is poor, as evidenced by significant swelling and gel formation after 7 h in a 1 M NaOH solution at 40 °C [102].

It was shown that introducing flexible side chains facilitates the formation of a microphase-separated structure, enhancing ion transport efficiency while suppressing water swelling in AEM and strengthening its alkaline stability [81,82]. Side-chain-type PPO anion exchange membranes with varying spacer lengths via a one-pot Wittig reaction were prepared successfully. The induced microphase separation from the spacer resulted in a membrane exhibiting low water swelling, high ion conductivity (99.5 mS/cm at 80 °C), and excellent chemical stability in an alkaline environment at 80 °C, especially at low IEC values [103]. This is attributed to the microphase-separated structure induced by the long side chains between the polymer main chain and the ion exchange groups.

Furthermore, introducing alkyl spacer groups between the polymer main chain and the cationic functional groups significantly enhances OH^-^ conductivity. When the IEC is equivalent, at 80 °C, the ion conductivity increases from 12 mS/cm to 64 mS/cm compared to AEM without spacer groups [104]. By regulating the length of the alkyl chain, it was found that, under similar IEC conditions, the ion conductivity is highest when the alkyl chain consists of 6 carbon atoms (62.7 mS/cm, 80 °C) [105]. This is because, with a shorter spacer, the mobility of the ion exchange groups is insufficient, making it difficult to form effective ion transport channels. On the other hand, when the spacer is too long, the decrease in hydrophilicity caused by the side chain is unfavorable for OH^-^ transport.

Block copolymer AEMs have received significant attention due to the fact that these membranes consist of two or more segments with different compositions and properties. The hydrophilic/hydrophobic differences between the segments facilitate the construction of efficient ion transport channels. Moreover, the longer the hydrophilic segment within the membrane, the more developed the ion transport channels formed, leading to higher conductivity [106].

**Figure 10:**
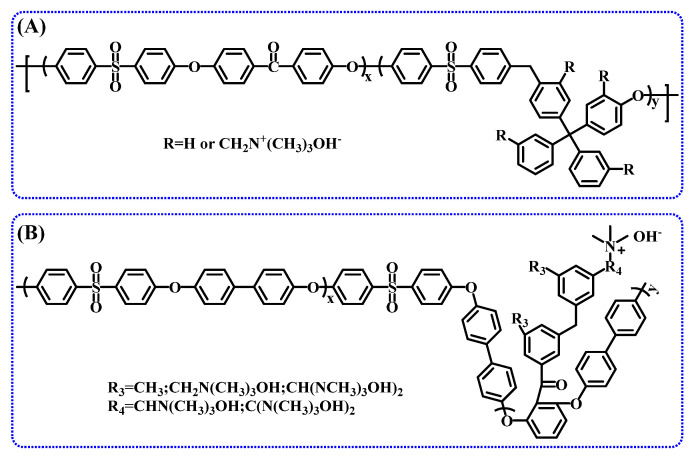
Block copolymer structure with a microphase-separated structure, fluorenyl-based multiblock AEM (**A**) and poly(arylene ether sulfone) block AEM (**B**).

Fluorine-terminated oligomers and hydroxyl-terminated oligomers were employed to prepare a multi-block AEM containing fluorenyl groups, and its chemical structure is shown in Figure 10 A. Due to its distinct microphase-separated structure, the ion conductivity of this membrane reaches up to 144 mS/cm at 80 °C (IEC = 1.93 meq/g), approximately 3.2 times higher than that of a random-type AEM with a similar IEC (IEC = 1.88 meq/g) [107]. Despite its higher conductivity, the membrane, with quaternary ammonium groups located at the benzyl position, is susceptible to attack and degradation by OH^-^, resulting in poor alkaline resistance. Moreover, a poly (arylene ether sulfone) block copolymer was synthesized via a series of reactions, including pre-condensation, block copolymerization, bromomethylation, and Menshutkin reactions (Structure B in Figure 10) and employed to prepare AEM. This membrane effectively avoids Hofmann elimination caused by β-H, thereby enhancing the stability of the membrane. It was demonstrated that the AEM based on this block copolymer exhibited a well-defined microphase-separated morphology, with the highest ion conductivity reaching 86.3 mS/cm [106].

Although block copolymer AEMs easily form well-defined microphase-separated structures, constructing efficient ion transport pathways, block copolymers are inherently polydisperse, making it generally challenging to precisely control the microstructure of AEMs. Additionally, block copolymer AEMs tend to have higher water uptake, resulting in lower mechanical performance [108].

In summary, enhancing the hydrophilic/hydrophobic contrast between the polymer main chain and cationic side chains, coupled with the introduction of flexible side chains, further increases the freedom of movement and aggregation of ion exchange groups. This enables the construction of well-defined ion transport pathways, ultimately leading to an improvement in the ion conductivity of AEMs.

### 2.4. Other Strategies to Enhance AEM Ion Conductivity

Organic–inorganic hybridization provides another viable strategy for enhancing the performance of AEM by generating highly conductive nanoclusters within the membrane [109]. AEMs prepared using well-conductive inorganic nanofillers not only improve ion conductivity but also enhance membrane thermal stability and mechanical properties. This causes organic–inorganic hybrid composite membranes to have broad application prospects in the field of water electrolysis.

Common inorganic nanomaterials include titanium dioxide (TiO_2_) [110,111], graphene oxide (GO) [112,113,114], carbon nanotubes (CNT) [115], and metal-organic frameworks (MOF) [116], among others. These nanomaterials are typically subjected to functionalization and modification treatments before blending into films with functionalized or non-functionalized polymer main chains. Due to the abundant active sites on the surface and internal pores of inorganic nanomaterials are conducive to functionalization, thereby effectively enhancing the conductivity of AEM [110].

It has been shown that blending core–shell nanoparticles composed of SiO_2_ and densely functionalized polystyrene (PS) (70 wt%) with a polysulfone matrix to form a film that results in an AEM with remarkably high ion conductivity (188.1 mS/cm, 80 °C). This is attributed to the spatial confinement effect of SiO_2_, which causes the abundant conductive groups to aggregate in the functionalized PS “shell,” forming continuous ion transport channels. However, due to the high filler loading, inorganic particles tend to agglomerate. With increasing filler content, the aggregation becomes more pronounced, leading to structural defects in the membrane and a sharp decrease in mechanical strength [117].

Therefore, while organic–inorganic hybridization can effectively enhance the performance of AEM, excessive addition of inorganic particles leads to agglomeration, disrupting the homogeneous structure of the membrane, causing the formation of pores, and in severe cases, resulting in the crossover of H_2_/O_2_ gases. Hence, strict optimization of the amount of inorganic filler is required when employing this method.

### 2.5. Mechanical Properties of AEM

Additionally, to balance the ion conductivity and dimensional stability of AEM, the use of crosslinking agents to create a crosslinked network structure within the polymer can restrain excessive swelling of AEM, effectively improving its mechanical strength. This method has gained attention due to its simple preparation.

Commonly used crosslinking agents include diamines [118], piperazine [100,118,119,120], dithiols [121,122], etc. A PSF anion exchange membrane crosslinked with 4,4-trimethylenedipiperidine (TMDP) was fabricated, and the effects of crosslinking on the stability were explored. In a comparative experiment on alkaline stability, this AEM demonstrated excellent stability in 1 M KOH aqueous solution at 60 °C for 15 days, while the non-crosslinked polysulfone AEM became very brittle within 24 h. Furthermore, the crosslinked AEM exhibited outstanding dimensional stability compared to the non-crosslinked PSF anion exchange membrane, with the tensile strength increasing from 22.98 MPa to 35.07 MPa, attributed to the formation of a dense internal network structure [123]. Therefore, crosslinking can be a viable strategy for improving polymer main chain defects, especially in terms of mechanical properties.

In one research, poly(ethylene-co-tetrafluoroethylene) copolymers were crosslinked with dithiols via UV-induced thiol-ene click reactions, followed by quaternization to prepare crosslinked AEMs. The results showed that the tensile strength of the crosslinked membrane with an IEC content of 2.68 mmol/g reached nearly 40 MPa, with a fracture elongation of 23% [121]. In another study, quaternized PPO-based AEMs prepared using the same method demonstrated chemical and dimensional stability. After soaking in 4 M NaOH at 80 °C for 500 h, the crosslinked membrane maintained significantly higher hydroxide ion conductivity compared to the uncrosslinked AEM (uncrosslinked AEM decreased by 73.1%, while crosslinked AEM decreased by 52%) [124]. To address the balance between ion conductivity and stability of AEMs, crosslinked AEMs were developed using poly(phenylene-co-benzimidazole) as the backbone and dithiols as crosslinking agents, exhibiting improved mechanical properties (tensile strength increased from 25.51 MPa to 39.76 MPa, fracture elongation increased from 13.81% to 18.34%) and dimensional stability (swelling ratio < 15%) [122]. The tensile strength was measured at 48.4 MPa, with a fracture elongation of 50.8%.

AEMs based on polybenzimidazole (PBI) crosslinked with polyvinylbenzyl chloride (PVBC) exhibited lower water uptake (48% at 80 °C) and swelling ratio (11% at 80 °C). The supporting effect of PBI and the crosslinking structure endowed the membrane with good mechanical properties (tensile strength of 37.5 MPa) [120]. Thus, crosslinking is an effective method to enhance the dimensional stability of AEMs. These data are summarized in Table 1.

In general, the poor alkaline stability and low ion conductivity of AEM are the main challenges for reducing energy losses and ohmic voltage drops in AEMWE. Thus, ether-free main chains are the preferred choice for AEM structure design, and selecting suitable functional groups to construct ion transport channels is an effective strategy to promote faster ion transport and enhance ion conductivity.

All the above discussions focus on the academic research works in the field of the development of AEMs. Further, exploring the perspective of already published patents on the most promising commercially viable AEM research directions would be equally important.

## 3. Progress in Patent Research of Anion Exchange Membranes

Anion exchange membranes have a wide range of applications, such as water purification processes [123,126], electrodialysis [127,128,129], biosensor [130], and water electrolysis [131,132]. The first anion exchange membrane was developed by scientists at the Tokuyama Soda Company in Japan using crosslinked divinylbenzene and trimethylamine quaternization of polyvinyl chloride [133]. The common method for preparing anion exchange membranes in patents is to introduce side chains containing cation exchange groups (such as ammonium, imidazolium, and quaternary ammonium groups) onto the polymer main chain via chemical or physical irradiation methods [134,135].

### 3.1. Composite Membranes

Composite membranes have become one of the main research topics due to their excellent properties against pristine membranes [136,137,138,139]. Typically, functional additives such as ion exchange polymers, metal oxides, graphene, carbon nanotubes, etc., are coated or impregnated into porous substrates [140,141] or nanofiber networks [141], such as polytetrafluoroethylene, poly (vinylidene fluoride), polyethylene, etc. Composite AEMs prepared by this method exhibit high ion conductivity, excellent size stability, and alkali resistance [142,143,144].

As of now, composite membranes made of organic polymers and inorganic fillers have been widely described in journals and patents [134,142,143,144,145,146,147]. As early as 1999, a patent described the blending of ionomer solutions with silicates to form a composite membrane [148]. By 2002, the first reports of organic phases being in an ionic state and composite membranes obtained via covalent crosslinking had emerged. The composite membrane, obtained by blending a metal salt (such as ZrOCl_2_) with a polymer solution containing crosslinking groups, exhibited excellent mechanical and thermal stability, as well as good ion conductivity [149].

In addition to polymer blending, electrospinning technology has also been applied to prepare AEMs. Two polymer fibers are electrospun separately to form nanofiber mats, and one of the polymer nanofiber mats is heated to fill the gaps in the other polymer nanofiber mat. This method avoids multiple impregnation steps for porous fiber and can fill the pores, but the membrane’s durability still needs improvement [150]. In a patent published in the same year, ion exchange polymers were impregnated into a nanofiber network [151]. The ion conductivity of this composite membrane exceeded 80 mS/cm, significantly enhancing the performance of AEMs and addressing the collapse/void issues of porous substrates.

**Figure 11:**
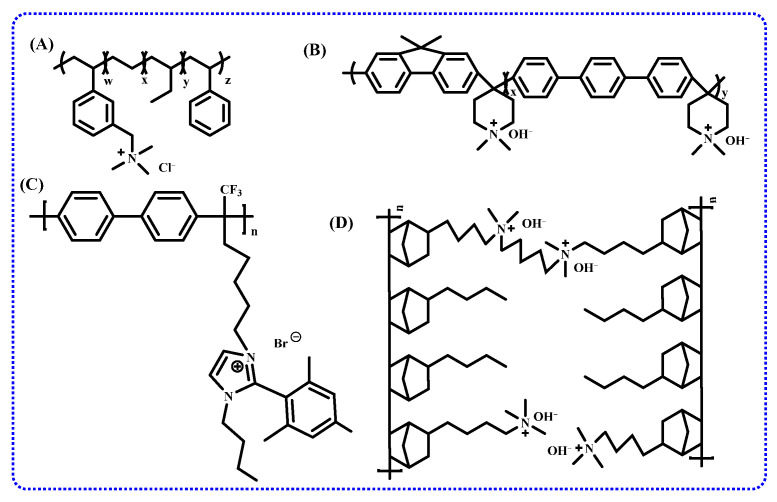
Main chain structures of anion exchange membranes in the patents, co-polymerized AEM (**A**), fluorene-based AEM (**B**), polycyclic aromatic alkyl main chain (**C**), and poly-cycloolefin block copolymer (**D**).

To improve alkaline stability, copolymerization has become a preferred research direction. For example, a triblock anion exchange membrane synthesized by copolymerizing styrene, ethylene, and propylene, followed by chloromethylation and quaternization, can be used in systems such as fuel cells, electrolysis, and flow batteries; the main chain structure is shown in Figure 11A [152]. Additionally, an AEM prepared from repeating units of vinylbenzyl trimethylammonium salt, styrene, and divinylbenzene exhibits good alkaline stability. Particularly, the addition of plasticizers, polyvinylidene fluoride, and other additives significantly enhances the ion exchange capacity, ion conductivity, mechanical properties, chemical properties, and processing performance of the anion exchange membrane while also offering cost reduction advantages [138].

### 3.2. Ether-Free Main Chains

Most commercially available AEMs are based on crosslinked polystyrene or copolymers of styrene and divinylbenzene. However, these materials lack sufficient stability at high pH values. In recently reported patents [153,154,155], some innovators have synthesized polymers with specific groups, such as polyfluorene or other non-ether polymers (Figure 11B), via condensation or super acid-catalyzed polyhydroxy alkylation reactions. Crosslinking is used to reduce membrane swelling and enhance mechanical stability, or stability of the cationic functional groups is improved via conjugation and electron-donating effects. A patent published in 2014 reveals that an anion exchange membrane, obtained by introducing N-vinylimidazole into a non-ether polymer main chain via radiation polymerization and quaternizing the side chains with halogenated alkane, demonstrates excellent alkaline stability by preventing nucleophilic substitution and elimination reactions [156]. Furthermore, the chemical stability and mechanical properties of the polyaromatic alkyl main chain or polyaromatic crown ether main chain have been confirmed in other patent studies (Figure 11C) [157]. In comparison to traditional AEMs, these non-ether main-chain AEMs exhibit lower water uptake and swelling and demonstrate excellent alkaline stability at high pH values.

The ideal characteristics of an AEM not only include excellent alkaline stability but also the presence of effective hydroxide ion transport channels. Despite significant progress in the past decade, there is still considerable room for development in the performance of AEMs compared to PEMs [158].

### 3.3. Microphase-Separated Structure

To promote the transport of hydroxide ions, the construction of microphase-separated structures has become a focus in many patents as an effective method to enhance hydroxide ion conductivity. It has been observed that the formation of block copolymers aids in achieving phase separation within the polymer, facilitating the creation of high-mobility ion transport channels within the membrane [159]. For example, a hydrophobic polysulfone main chain and a hydrophilic polyethylene oxide undergo simultaneous quaternization reactions, creating a phase-separated structure with both hydrophilic and hydrophobic regions within the membrane. This formation results in a double continuous region that constitutes ion transport channels, facilitating the transport of ions [157]. Furthermore, in a patent disclosed in 2022, a block copolymer of poly-cycloolefin (Figure 11D) synthesized from various functionalized norbornene monomers exhibited remarkable ion conductivity (198 mS/cm at 80 °C) [160]. However, compared to non-block copolymers, the synthesis of block copolymers is more challenging and complex.

Designing polymer materials into a comb-like structure, where pendant side chains are connected to cationic functional groups, facilitates the construction of a hydrophilic/hydrophobic microphase-separated structure with a simple synthesis method. In the current patent [161], it has been observed that the ion conductivity of the comb-like PBI anion exchange membrane obtained via this method reaches 76.3 mS/cm at 80 °C. Although this PBI anion exchange membrane exhibits excellent conductivity, its water uptake is as high as 298.5%, severely impacting the size stability of the membrane.

As discussed, besides alkaline stability and high ion conductivity, maintaining mechanical integrity is crucial, and mild crosslinking helps control water uptake and enhance mechanical stability. In several patents, AEMs crosslinked via physical or chemical means demonstrate exceptionally high mechanical and chemical stability. For example, covalent crosslinking using polyethylene glycol terminated with epoxy groups reduces swelling, thereby improving mechanical stability [162]. Alternatively, films of poly(acrylamide-co-dimethylallyl chloride)-crosslinked with glutaraldehyde, about 20 μm thick, exhibit stable chemical and mechanical properties [163]. Interestingly, in composite AEMs composed of a surface layer with a crosslinked structure and an anion exchange membrane matrix, when the crosslinked portion carries an opposite charge to the cationic functional groups of the ion exchange membrane, it not only enhances the stability of the AEM but also improves the membrane’s ion selectivity, attributed to the charge repulsion at the surface layer [164].

Crosslinking is considered a direct approach to improving the mechanical and physicochemical properties of anion exchange membranes [162,165,166,167,168]. High mechanical stability is particularly crucial for thin (<50 μm) AEMs and AEM water electrolysis operations under high-pressure differentials. However, improper application of crosslinking may lead to deteriorating AEM performance. For instance, the use of long-chain crosslinking agents can induce AEM crystallization, thereby compromising various physicochemical properties, such as reducing hydrophilicity [169].

Nevertheless, AEMs still face major challenges, such as hydrogen production efficiency, long-term stability, and cost-effectiveness under operating conditions. Therefore, non-ether main chains and suitable functional groups are the preferred choices to enhance stability, and constructing a microphase-separated structure to form continuous ion transport channels is one of the most effective strategies to improve ion conductivity.

## 4. Conclusions and Outlook

In summary, to address the key factors limiting large-scale electrolytic hydrogen production, such as low hydrogen production efficiency, poor long-term stability, and low ion conductivity of AEMs in water electrolysis, this review provides an overview of recent research progress in anion exchange membranes. It focuses on analyzing methods to enhance AEM ion conductivity and alkaline stability. Additionally, by examining patents and the current application status of AEMs, the review identifies challenges and potential solutions in practical AEM water electrolysis production, aiming to achieve efficient and green hydrogen production.

Thanks to the current ideas, AEM has made significant progress and has achieved substantial advancements in various dimensions, ranging from molecular design to laboratory-scale trials. High ion conductivity exceeding 100 mS/cm and alkaline stability exceeding 1000 h have been achieved [170]. The future trends of AEM are becoming evident, although the development of hydrogen production at the pilot scale is still in progress. The main challenges for AEM currently are alkaline stability and ion conductivity.

Several potential future trends in AEM design have emerged:

1. Ether-free polyaromatic main chains and N-cyclic quaternary ammonium are expected to meet the stability requirements of AEM.
2. Systematic studies of microphase separation structures at the molecular level, using molecular simulations to predict substance transport within the membrane, are likely to advance AEM development.
3. AEM still requires sufficient ion exchange capacity to achieve high-performance AEMWE, and the reliability of hydrophilic/hydrophobic microphase separation structures remains crucial.

Achieving high-performance AEM materials is still in the early stages, and further developments in pilot-scale hydrogen production are needed. Subsequent efforts will focus on advancing large-scale processing and low-cost manufacturing to meet the applications in global energy systems.

## Figures and Tables

**Table 1:** Comparison of performance of AEMs reported in the literature.

Name	Type	IEC (mmol/g)	Tensile Strength (MPa)	Water Uptake (%)	Swelling Ratio (%)	Ref.
CAPSF-5	uncrosslinked	1.92	22.98	13.04	9.42	[123]
Neosepta AMX	crosslinked	2.16	35.07	44.23	4.22	[123]
V-2-H-1	crosslinked	2.93	~40 MPa	<60 (90 °C)	<15	[121]
X60Y30C6	crosslinked	2.38	-	93 (80 °C)	35	[124]
PcPBI-Nb-2.33	uncrosslinked	2.37	25.51	83.0 (80 °C)	6.9	[122]
PcPBI-Nb-C2	crosslinked	2.25	39.76	100.3 (80 °C)	5.9	[122]
PBI-PVBC-NMPD/OH	crosslinked	2.31	37.5	48 (80 °C)	11	[120]
PTPBHIN-O_19_	crosslinked	1.64	64.8	133 (80 °C)	10.53	[125]
m-TPNPiQA	uncrosslinked	2.54	<20	65.2 (80 °C)	25.7	[100]
C-IL-100	crosslinked	2.99	22.91	97.0	35.9	[100]

## Data Availability

No new data were created or analyzed in this study. Data sharing is not applicable to this article.
